# Detection and Genetic Characterization of *Enterocytozoon hepatopenaei* in Giant Freshwater Prawn (*Macrobrachium rosenbergii*) Imported into South Korea

**DOI:** 10.3390/ani15223286

**Published:** 2025-11-13

**Authors:** Hye Jin Jeon, Bumkeun Kim, So Young Bang, Yukyung Kim, Jee Youn Hwang, Patharapol Piamsomboon, Ji Hyung Kim, Jee Eun Han

**Affiliations:** 1 Laboratory of Aquatic Biomedicine, College of Veterinary Medicine, Kyungpook National University, Daegu 41566, Republic of Korea; jhj1125@knu.ac.kr (H.J.J.); aam_kim@knu.ac.kr (B.K.); bsy950218@knu.ac.kr (S.Y.B.); ygim9904@knu.ac.kr (Y.K.); 2 Institute for Veterinary Biomedical Science, Kyungpook National University, Daegu 41566, Republic of Korea; 3 Quarantine and Inspection Division, National Fishery Products Quality Management Service, 337, Haeyang-ro, Yeongdo-gu, Busan 49111, Republic of Korea; jinihwang@korea.kr; 4 Department of Veterinary Medicine, Faculty of Veterinary Science, Chulalongkorn University, Bangkok 10330, Thailand; patharapol.p@chula.ac.th; 5 Department of Food Science and Biotechnology, College of Bionano Technology, Gachon University, Seongnam 13120, Republic of Korea

**Keywords:** crustaceans, internal transcribed spacer-1, spore wall protein, shrimp, small subunit ribosomal RNA, trading

## Abstract

**Simple Summary:**

*Enterocytozoon hepatopenaei* (EHP) is an intracellular parasite that causes substantial economic losses to the global shrimp industry. Although penaeid shrimps are particularly vulnerable to EHP infection and have been extensively studied, *Macrobrachium rosenbergii* (giant freshwater prawn), another important species widely farmed in warm-climate regions, is also affected by this pathogen. Despite the increasing global incidence of EHP infection in *M. rosenbergii*, studies on this species remain limited compared to those on penaeid shrimps. Here, we monitored the EHP infection in *M. rosenbergii* imported from India and Vietnam to South Korea and conducted genetic analyses to better characterize its genetic diversity.

**Abstract:**

This study investigated *Macrobrachium rosenbergii* imported from India (15 batches, *N* = 180) and Vietnam (7 batches, *N* = 84) between 2023 and 2024, for *Enterocytozoon hepatopenaei* (EHP) monitoring and genetic analysis. Polymerase chain reaction assays detected EHP in 13.3% (2/15) and 71.4% (5/7) samples from India and Vietnam, respectively. The sequence of the small subunit ribosomal ribonucleic acid region of the EHPs isolated from *M. rosenbergii* showed no significant differences from those available in GenBank. Interestingly, spore wall protein (SWP) 1 region analysis revealed that *M. rosenbergii* EHPs could be divided into three groups, some of which were closely related to *Penaeus vannamei* EHPs. Similarly, the internal transcribed spacer-1 (ITS-1) region analysis divided *M. rosenbergii* EHPs into two groups, with some showing close relationships with *P. vannamei* EHPs. Phylogenetic analyses based on the SWP 1 and ITS-1 regions suggested that EHPs infecting *M. rosenbergii* exhibited greater genetic diversity than those infecting *P. vannamei*. This study provides the first report of EHP detection in *M. rosenbergii* imported from India and Vietnam to South Korea. Further genome-based analyses are necessary for a comprehensive genetic characterization of EHPs infecting *M. rosenbergii* from various geographical regions.

## 1. Introduction

Microsporidia are intracellular parasites that infect various hosts and produce environmentally resistant spores [1]. Their spores have a thick-layered wall, which enables them to survive for extended periods under harsh environmental conditions, such as high temperatures [2]. The spore wall is composed of an outer exospore and an inner endospore made of chitin and protein [2]. When a spore is exposed to environmental stimuli, it can increase the internal osmotic pressure, cause swelling and eventually injecting the polar filament into a host cell [3]. To date, over 50 genera of microsporidia have been identified in aquatic arthropods, including 20 genera that infect crustaceans belonging to the class Malacostraca, including shrimp, crabs, and lobsters [4].

Among these, *Enterocytozoon hepatopenaei* (EHP) causes substantial economic losses to shrimp farming [5]. EHP infection is mostly asymptomatic, making early detection difficult and leading to its spread across farms. In shrimp, this microsporidium proliferates in the cytoplasm of hepatopancreatic tubular epithelial cells, leading to growth retardation and size variation among individuals [5,6,7,8]. Notably, although EHP infection alone rarely causes high mortality, co-infection with other pathogens such as bacteria and viruses can significantly increase mortality rates and exacerbate symptoms [9,10]. EHP infection was initially reported in penaeid shrimps, including *Penaeus monodon* [9], *Penaeus vannamei* [11], and *Penaeus stylirostris* [12]; however, it has more recently been detected in *M. rosenbergii* in China [13], South Korea [14], and Japan (2023, available in the GenBank database).

*Macrobrachium rosenbergii* (commonly known as giant freshwater prawn) is the largest freshwater prawn species worldwide and widely farmed in various regions across the globe, including Northwest India, Bangladesh, China, Indonesia, Thailand, Vietnam, the Philippines, Northern Australia, and South Korea [15,16,17]. Since 1980, global production has increased significantly, exceeding 220,000 tons in 2020 [18]. In South Korea, over 47 tons of *M. rosenbergii* was imported from major prawn-producing countries between 2019 and 2023 [19].

The genetic diversity of EHP and its potential for horizontal transmission across various shrimp species highlight the need for molecular characterization to develop effective management strategies for EHP. This study aimed to highlight the potential risk of introducing EHPs into South Korea via imported shrimp, for which EHP monitoring is currently not conducted. In this study, we investigated the prevalence and genetic diversity of EHP in imported *M. rosenbergii* by analyzing the small subunit ribosomal RNA (SSU rRNA) and spore wall protein (SWP) 1 regions of EHP. Phylogenetic analyses were performed using these sequences for comparison with EHP strains available in the GenBank database.

## 2. Materials and Methods

### 2.1. Sample Collection

A total of 22 batches (12 prawns per batch) of frozen *M. rosenbergii* imported from India (*N* = 15 batches) and Vietnam (*N* = 7 batches) were collected from local retail markets in South Korea. All samples were transported to the laboratory on ice and stored at −80 °C until further analysis. Detailed information on the *M. rosenbergii* samples is presented in Table 1.

### 2.2. DNA Extraction and EHP Monitoring

From each batch, *M. rosenbergii* (*N* = 10) was randomly selected, and hepatopancreatic tissues were pooled as one sample. DNA was extracted from 30 mg hepatopancreatic tissue using the DNeasy Blood & Tissue Kit (Qiagen, Hilden, Germany) according to the manufacturer’s instructions. EHP was detected by PCR using the 510-F/510-R primer pair, targeting the SSU rRNA [12] (Table 2).

### 2.3. Sequence Analysis of SSU rRNA, SWP 1, and Internal Transcribed Spacer-1 Regions of EHP

EHP-positive samples (identified using the 510-F/510-R primer pair) were further analyzed by sequencing the SSU rRNA [11], SWP 1 region [13], and internal transcribed spacer-1 (ITS-1) regions [21] (Table 2). The resulting amplicons were sequenced at Bioneer (Daejeon, Republic of Korea). The SSU rRNA, SWP 1, and ITS-1 sequences obtained in this study were deposited in the GenBank database (Supplementary Table S1). The obtained sequences were compared to other sequences listed in GenBank using BLASTn (ver. 2.17.0) to confirm the identity of the EHP sequences. Pairwise distances between the obtained sequences and reference EHP sequences derived from the GenBank database were compared using Geneious Prime (ver. 2024; http://www.geneious.com).

### 2.4. Phylogenetic Tree Analysis

Phylogenetic analyses were conducted using SSU rRNA, SWP 1, and ITS-1 sequences from EHP strains available in the GenBank database. (1) SSU rRNA: *P. vannamei* EHP (China, KX981865; India, KY643648; Republic of Korea, MZ819965; Vietnam, KP759285) and *M. rosenbergii* EHP (Republic of Korea, OP363710); (2) SWP 1: *P. vannamei* EHP (India, MH365434; Indonesia, KY593133; Republic of Korea, MZ541056; Thailand, MG015710), *M. rosenbergii* EHP (China, MW269619); and (3) ITS-1: *P. vannamei* EHP (China, OR162445 and OR168076; South Korea, ON015652; Thailand, MNPJ00000000) (all sequences were retrieved from the GenBank database: http://www.ncbi.nlm.nih.gov/genbank, accessed on 25 July 2024). The sequences were initially aligned using ClustalW (ver. 2.1), and non-overlapping genomic regions at the fragment ends were trimmed. The trimmed sequences were used for phylogenetic analyses. Phylogenetic trees based on the SSU rRNA, SWP 1, and ITS-1 sequences of EHP strains were constructed using the maximum-likelihood method with 1000 bootstrap replications implemented in MEGA X (version 11.0.13) [22].

## 3. Results

### 3.1. EHP Monitoring in Imported M. rosenbergii

PCR analysis identified EHP in two *M. rosenbergii* samples from India (23-026C6-2-INDIA and 23-026C7-2-INDIA; 2/15, 13.3%) and five *M. rosenbergii* samples from Vietnam (23-026C6-1-VIE, 23-026C7-1-VIE, 23-026C7-3-VIE, 23-026C9-2-VIE, and 23-026C11-1-VIE; 5/7, 71.4%) (Table 1).

### 3.2. Sequence Analysis of SSU rRNA, SWP 1, and ITS-1 Regions of EHP

SSU rRNA sequences were successfully obtained from six EHP-positive *M. rosenbergii* samples, except 23-026C11-1-VIE, which exhibited weak PCR amplification result. BLASTn searches revealed that the six sequences shared over 99.0% identity (query cover = 100%) with *P. vannamei* EHPs (originating from China, India, Republic of Korea, and Vietnam) and *M. rosenbergii* EHP (originating from Republic of Korea). The results of the pairwise distance analysis between the obtained six *M. rosenbergii* EHPs in this study and reference *P. vannamei* and *M. rosenbergii* EHPs available in the GenBank database are shown in Supplementary Table S2.

SWP 1 region sequences were successfully obtained from all EHP-positive *M. rosenbergii* samples. BLASTn searches revealed that two samples (23-026C7-2-INDIA and 23-026C7-3-VIE) shared 99.44% identity (query cover = 100%) with *P. vannamei* EHPs (originating from India and Thailand) and 92.05% identity (query cover = 98%) with *M. rosenbergii* EHP (originating from China). The remaining five samples (23-026C6-1-VIE, 23-026C6-2-INDIA, 23-026C7-1-VIE, 23-026C9-2-VIE, and 23-026C11-1-VIE) showed 97.78% identity (query cover = 100%) with *P. vannamei* EHPs (originating from India and Thailand) and 92.61% identity (query cover = 98%) with *M. rosenbergii* EHP (originating from China). The results of pairwise distance analysis between the seven *M. rosenbergii* EHPs obtained in this study and reference *P. vannamei* and *M. rosenbergii* EHPs available in GenBank are presented in Supplementary Table S3.

The ITS-1 region sequences were successfully obtained from seven EHP-positive *M. rosenbergii* samples. BLASTn searches revealed that two samples (23-026C7-2-INDIA and 23-026C7-3-VIE) had 93.51% identity (query cover = 90.0%), and the other five samples (23-026C6-1-VIE, 23-026C6-2-INDIA, 23-026C7-1-VIE, 23-026C9-2-VIE, and 23-026C11-1-VIE) had 80.76% identity (query cover = 100%) with *P. vannamei* EHPs (originating from China and South Korea). Pairwise distance analysis results between the seven *M. rosenbergii* EHPs obtained in this study and the reference *P. vannamei* EHPs available in the GenBank database are shown in Supplementary Table S4.

### 3.3. Phylogenetic Analysis

**Figure 1:**
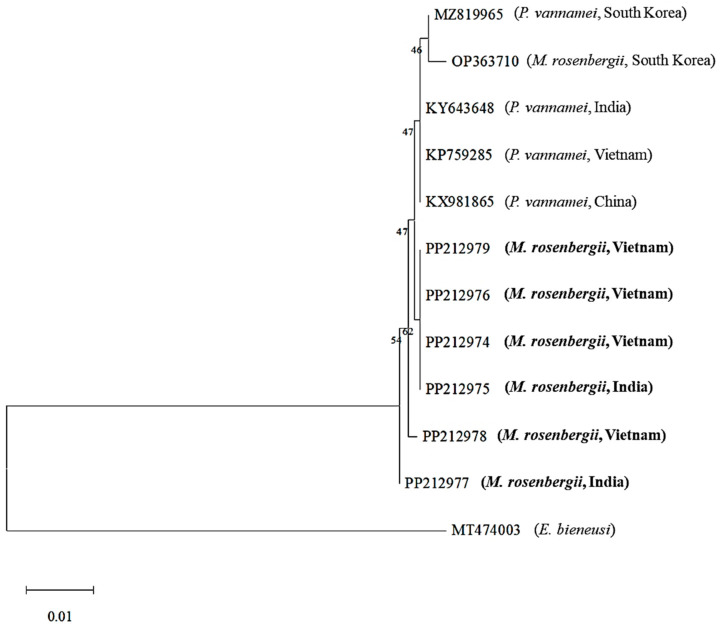
Maximum-likelihood phylogenetic tree based on nucleotide sequences of the small subunit ribosomal RNA (SSU rRNA) region of *Enterocytozoon hepatopenaei* (EHP), including *Macrobrachium rosenbergii* EHPs obtained in this study (in bold; GenBank accession Nos. PP212974, PP212975, PP212976, PP212977, PP212978, and PP212979). *Enterocytozoon bieneusi* (GenBank accession No. MT474003; family *Enterocytozoonidae*; order *Enterocytozoonida*) was used as an outgroup. Numbers at the branches indicate bootstrap values obtained using 1000 replicates. The scale bars represent 0.01 nucleotide substitutions per site. The trimmed alignment length was 778 bp.

Phylogenetic analysis based on SSU rRNA sequences was performed using the six *M. rosenbergii* EHP sequences obtained in this study; previously reported *P. vannamei* EHP sequences (*N* = 4) from China, India, South Korea, and Vietnam; and previously reported *M. rosenbergii* EHP sequence (*N* = 1) from South Korea available in the GenBank database. The resulting phylogenetic tree based on SSU rRNA sequences revealed that the *M. rosenbergii* EHPs from this study grouped closely with previously reported *P. vannamei* and *M. rosenbergii* EHPs (Figure 1).

**Figure 2:**
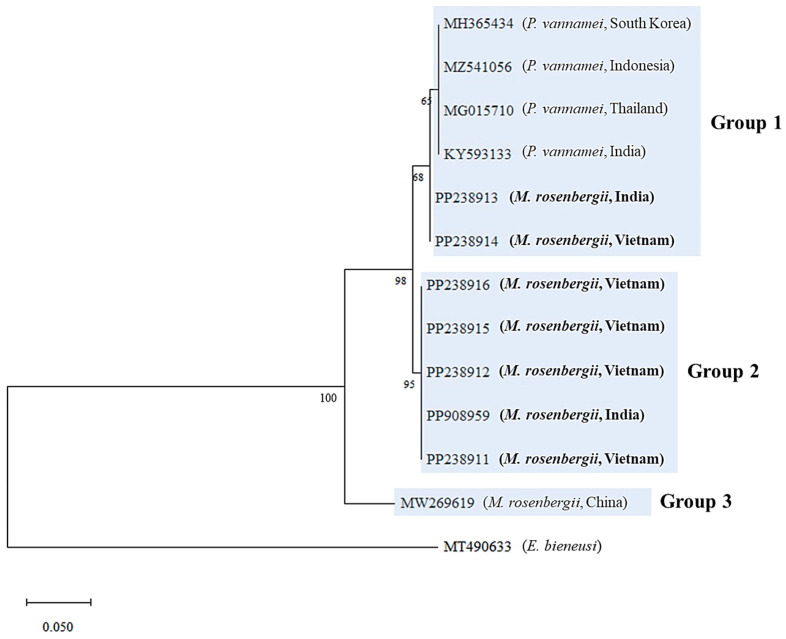
Maximum-likelihood phylogenetic tree based on nucleotide sequences of the spore wall protein (SWP) 1 region of *Enterocytozoon hepatopenaei* (EHP), including *Macrobrachium rosenbergii* EHPs obtained in this study (in bold; GenBank accession Nos. PP908959, PP238911, PP238912, PP238913, PP238914, PP238915, and PP238916). *Enterocytozoon bieneusi* (GenBank accession No. MT490633) was used as an outgroup. Numbers at the branches indicate bootstrap values obtained using 1000 replicates. The scale bars represent 0.05 nucleotide substitutions per site. The trimmed alignment length was 151 bp.

Phylogenetic analysis of the SWP 1 region was conducted using seven *M. rosenbergii* EHP sequences from this study; *P. vannamei* EHP sequences (*N* = 4) from India, Indonesia, South Korea, and Thailand; and an *M. rosenbergii* EHP sequence (*N* = 1) from China, available in the GenBank database. The tree based on the SWP 1 region was divided into three groups (Groups 1–3; Figure 2). Group 1 included two *M. rosenbergii* EHPs identified in this study (23-026C7-2-INDIA and 23-026C7-3-VIE), which were grouped with previously reported *P. vannamei* EHPs from Asian countries. Group 2 comprised the remaining five *M. rosenbergii* EHPs from this study (23-026C6-1-VIE, 23-026C6-2-INDIA, 23-026C7-1-VIE, 23-026C9-2-VIE, and 23-026C11-1-VIE), whereas Group 3 contained the previously reported *M. rosenbergii* EHP from China [13] (Figure 2 and Supplementary Figure S1).

**Figure 3:**
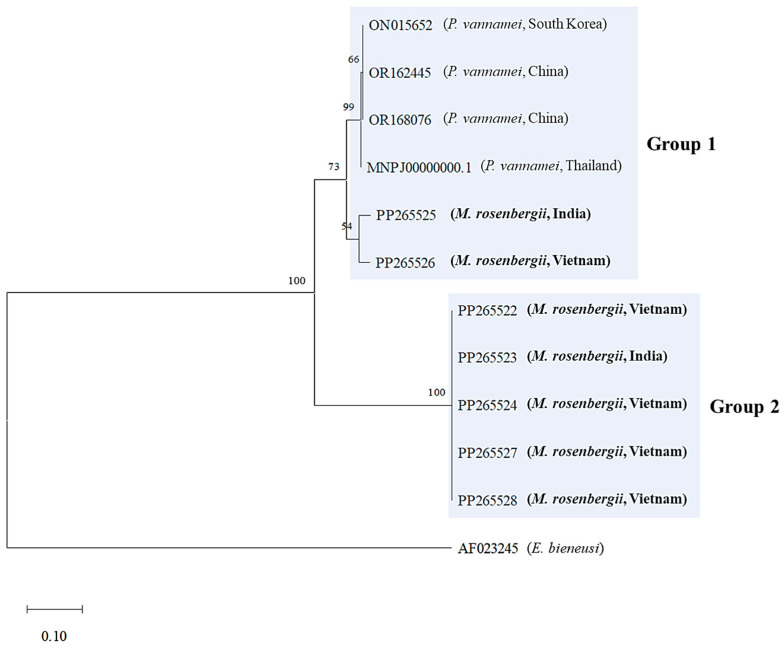
Maximum-likelihood phylogenetic tree based on the nucleotide sequences of the internal transcribed spacer-1 (ITS-1) region of *Enterocytozoon hepatopenaei* (EHP), including *Macrobrachium rosenbergii* EHPs obtained in this study (in bold; GenBank accession Nos. PP265522, PP265523, PP265524, PP265525, PP265526, PP265527, and PP265528). *Enterocytozoon bieneusi* (GenBank accession No. AF023245) was used as an outgroup. Numbers at the branches indicate bootstrap values obtained using 1000 replicates. The scale bars represent 0.10 nucleotide substitutions per site. The trimmed alignment length was 305 bp.

Phylogenetic analysis of the ITS-1 region was performed using the seven *M. rosenbergii* EHP sequences from this study, along with *P. vannamei* EHP sequences (*N* = 4) from China, South Korea, and Thailand, available in the GenBank database. The phylogenetic tree based on the ITS-1 region is divided into two groups (Groups 1 and 2), with identical compositions in the SWP 1 region (Figure 3). Group 1 included two *M. rosenbergii* EHPs identified in this study (23-026C7-2-INDIA and 23-026C7-3-VIE), which were grouped with previously reported *P. vannamei* EHPs from Asian countries. Group 2 comprised the remaining five *M. rosenbergii* EHPs (23-026C6-1-VIE, 23-026C6-2-INDIA, 23-026C7-1-VIE, 23-026C9-2-VIE, and 23-026C11-1-VIE) (Figure 3 and Supplementary Figure S2). This grouping pattern of *M. rosenbergii* EHPs in this study was similar to that observed in the SWP 1 region analysis, suggesting consistent genetic grouping.

## 4. Discussion

EHP infection in *M. rosenbergii* has been increasingly reported worldwide, including China [13], Republic of Korea [14], and Japan (OQ860232, retrieved from GenBank). EHP infections in *M. rosenbergii* are similar to those in *P*. *vannamei*, typically showing no noticeable clinical signs except growth retardation [14,23,24]. Histopathological changes such as inflammation, hemocytic infiltration, and tubular epithelial cell sloughing in the hepatopancreas are common [25], making EHP infections easy to overlook, often resulting in significant yield loss [26]. A previous study [27] has emphasized the importance of quarantining EHP in crustaceans imported to Republic of Korea. However, currently, no regulatory guidelines for EHP in the crustacean trade are available, and EHP is not listed as a quarantined infectious disease for imported shrimp either in Republic of Korea or by the World Organisation for Animal Health [28]. Furthermore, despite the recent global increase in EHP infections in *M. rosenbergii*, research on this issue has been limited compared to studies on EHP in penaeid shrimp.

In the present study, we confirmed the presence of EHP in frozen *M. rosenbergii* imported from India and Vietnam to Republic of Korea. Interestingly, EHP was not detected in samples collected in 2024, which may reflect the increased global awareness of the risk of EHP. Strict controls implemented by producing countries after the WOAH classified EHP as an emerging disease are likely to contribute to the declining prevalence. However, to the best of our knowledge, this is the first report of EHP detection in *M. rosenbergii* imported from these countries to Republic of Korea. Additionally, we analyzed the nucleotide sequence and phylogenetic tree of three regions (SSU rRNA, SWP 1, and ITS-1) to understand the genetic characteristics of *M. rosenbergii* EHPs. The SSU rRNA region is highly conserved and has been used to accurately assess the evolutionary relationships among EHP isolates, as the SWP 1 and ITS-1 regions have high sequence variability and have been used for species-level identification and analysis of genetic diversity within species. Although the SSU rRNA sequences of the EHP-positive *M. rosenbergii* showed high nucleotide identities (≥99.4%) with previously reported EHP sequences, more variable regions, SWP 1, and ITS-1 revealed phylogenetic differentiation.

Phylogenetic analysis of the SWP 1 region divided into three groups (Groups 1, 2, and 3) and seven *M. rosenbergii* EHPs formed two distinct groups (Groups 1 and 2). Notably, among the seven *M. rosenbergii* EHPs, two (23-026C7-2-INDIA: PP238913 and 23-026C7-3-VIE: PP238914) were closely related to *P. vannamei* EHPs from Asian countries (Group 1), whereas the remaining five were grouped into another group (Figure 2). This conflicts with the findings by Wang et al. (2023), who reported that *M. rosenbergii* EHPs are genetically distinct from *P. vannamei* EHPs in Asian countries [13]. In addition, the seven *M. rosenbergii* EHPs were distinct from the previously reported Chinese *M. rosenbergii* EHP (Group 3, Figure 1). The ITS-1 region, known for its genetic diversity, is commonly used for genotyping and diagnosing microsporidia [21,29,30]. In this study, we obtained seven ITS-1 sequences from *M. rosenbergii* EHPs. Similar to the SWP 1 phylogeny, two EHPs (23-026C7-2-INDIA: PP265525 and 23-026C7-3-VIE: PP265526) were grouped with *P. vannamei* EHPs from Asian countries (Group 1), whereas the remaining five were distinct from Group 1 and formed Group 2 (Figure 3). The heterogeneity within *M. rosenbergii* observed in the SWP 1 and ITS-1 analyses in this study suggests that *M. rosenbergii* EHPs can be classified into two groups: those closely related to *P. vannamei* EHPs and those that are genetically distinct. Interestingly, the samples from India and Vietnam were grouped in the same branch. Therefore, genetic variation was not clearly related to origin. Moreover, shrimp size had no clear relationship with genetic variation, as shrimp of different sizes were included in the same group. Whether the phylogenetic similarities shown in the SWP 1 and ITS-1 regions also affect the virulence factors of EHP remains unclear. Nevertheless, two factors (genetic similarity between *M. rosenbergii* EHP and *P. vannamei* EHP and genetic diversity within *M. rosenbergii* EHP) suggest genomic divergence within EHP species and a possible host range expansion, encompassing both marine and freshwater environments. The adaptation of the pathogen to various environments and many other species raises concerns about disease quarantine and management.

The global trade of crustaceans has created opportunities for spreading pathogens such as EHP across borders. EHP can adapt to various crustacean hosts and potentially be transmitted for up to 14 days under freezing conditions [31]. Therefore, effective EHP monitoring is essential for both global crustacean trade and domestic aquaculture. Although conventional PCR is commonly used for EHP detection, strict surveillance using quantitative methods, such as qPCR or digital PCR, is necessary for accurate management, which can significantly improve the aquaculture industry. While this study focused primarily on the genetic analysis of *M. rosenbergii* EHPs, further research is needed to examine the transferability between crustacean species and pathogen activity under varying conditions, such as temperature, salinity, and pH, to clearly understand its infectivity and pathogenicity. In addition, genetic diversity may reflect differences in origin or sample variation, suggesting that further investigation with broad sampling is required. Our findings contribute to a comprehensive understanding of *M. rosenbergii* EHP and provide a foundation for further epidemiological studies, transmission route investigation, and improved EHP infection management in *M. rosenbergii*.

## 5. Conclusions

This study monitored EHP occurrence in 22 frozen *M. rosenbergii* samples (10 shrimp from each batch were pooled and treated as one sample) imported from India (*N* = 15) and Vietnam (*N* = 7) to Republic of Korea, via PCR assays. The genes in SSU rRNA, SWP 1, and ITS-1 regions of EHP-positive samples were analyzed. The genetic analysis of the SSU rRNA region revealed that the *M. rosenbergii* EHPs identified in this study were nearly identical (≥99.4%) to those available in the GenBank database. However, the sequence of SWP 1 and ITS-1 regions varied, where phylogenetic analysis separated *M. rosenbergii* EHPs into two distinct groups: one closely related to *P. vannamei* EHPs and the other genetically distinct. These findings, along with the genetic similarity between *M. rosenbergii* EHP and *P. vannamei* EHP and genetic diversity within *M. rosenbergii* EHP, indicate genomic divergence within EHP species and a possible expansion of the host range, encompassing both marine and freshwater environments. This is the first report of EHP detection in *M. rosenbergii* imported from India and Vietnam to Republic of Korea. These findings highlight the need for stringent monitoring of EHP in *M. rosenbergii* before global trade as well as the establishment of guidelines for controlling EHP infection.

## Figures and Tables

**Table 1:** Sample information and PCR results.

Sample ID	Country	Collection Month/Year	Length (cm)	Weight (g)	EHP Detection ^1^
23-026C5-1-VIE	Vietnam	May 2023	20.0–23.0	121.1–157.7	−
23-026C6-1-VIE	Vietnam	June 2023	14.0–18.3	33.0–60.0	+
23-026C6-2-INDIA	India	June 2023	20.5–23.0	93.6–153.4	+
23-026C7-1-VIE	Vietnam	July 2023	15.0–18.6	49.5–89.4	+
23-026C7-2-INDIA	India	July 2023	19.0–22.5	105.5–183.6	+
23-026C7-3-VIE	Vietnam	July 2023	14.4–18.0	31.4–75.8	+
23-026C8-1-INDIA	India	August 2023	20.9–26.1	95.1–199.4	−
23-026C8-2-VIE	Vietnam	August 2023	14.7–16.3	51.8–80.5	−
23-026C9-1-INDIA	India	September 2023	19.0–22.8	92.6–130.7	−
23-026C9-2-VIE	Vietnam	September 2023	15.0–16.4	45.7–77.8	+
23-026C10-1-INDIA	India	October 2023	19.5–22.8	90.6–140.2	−
23-026C11-1-VIE	Vietnam	October 2023	15.8–18.5	43.4–79.0	+
24-004C2-1-INDIA	India	February 2024	18.9–21.4	96.1–161.6	−
24-004C2-2-INDIA	India	February 2024	19.0–21.4	97.4–159.0	−
24-004C3-1-INDIA	India	March 2024	20.2–22.7	110.6–159.4	−
24-004C3-2-INDIA	India	March 2024	19.2–22.9	101.3–137.6	−
24-004C4-1-INDIA	India	April 2024	18.4–22.1	76.9–139.6	−
24-004C4-2-INDIA	India	April 2024	18.9–21.9	87.5–143.9	−
24-004C4-3-INDIA	India	April 2024	23.3–26.6	185.0–289.9	−
24-004C5-1-INDIA	India	May 2024	22.5–25.5	95.5–143.0	−
24-004C5-2-INDIA	India	May 2024	19.4–21.9	86.8–134.5	−
24-004C6-1-INDIA	India	June 2024	22.2–25.2	109.56–148.15	−

^1^ For EHP detection, the 510-F/R primer set was used [6]. +: positive, −: negative.

**Table 2:** Primer information used in this study.

Target	Primer	Sequence (5′ to 3′)	Amplicon Size (bp)	Reference
SSU rRNA ^1^	510-F	GCCTGAGAGATGGCTCCCACGT	510	[12]
	510-R	GCGTACTATCCCCAGAGCCCGA		
SSU rRNA ^2^	18S-F	CACCAGGTTGATTCTGCCTGA	1146	[12]
	18S-R	TCTGAAATAGTGACGGGCGG		
SWP 1	1F (1st)	TTGCAGAGTGTTGTTAAGGGTTT	514	[20]
	1R (1st)	CACGATGTGTCTTTGCAATTTTC		
	2F’ (2nd)	GCAGAGTGTTGTTAAGGGTTTAAG	182	[13]
	2R’ (2nd)	GCTGTTTGTCWCCAACTGTATT		
ITS-1	ITS1-1F (1st)	CGCCCGTCACTATTTCAGAT	603	[21]
	ITS1-1R (1st)	TACGTTCGTCATCGCTGCTA		
	ITS1-2F (2nd)	GAACCTGCTGTGGGATCATT	400	
	ITS1-2R (2nd)	AATTTTTGCTTGGCTCATTCT		

^1^ For EHP monitoring. ^2^ For sequence analysis.

## Data Availability

The original contributions presented in this study are included in the article/Supplementary Materials. Further inquiries can be directed to the corresponding authors.

## Supplementary Materials

The following supporting information can be downloaded at: https://www.mdpi.com/article/10.3390/ani15223286/s1, Table S1: Detailed information on the SSU rRNA, SWP 1, and ITS-1 sequences of *Enterocytozoon hepatopenaei* (EHP) obtained from this study; Table S2: Nucleotide sequence identities of the SSU rRNA region among *M. rosenbergii* EHPs obtained in this study and other EHPs available in the GenBank database; Table S3: Nucleotide sequence identities of the SWP 1 region among *M. rosenbergii* EHPs obtained in this study and other EHPs available in the GenBank database; Table S4: Nucleotide sequence identities of the ITS-1 region among *M. rosenbergii* EHPs obtained in this study and other EHPs available in the GenBank database; Figure S1: Alignment of nucleotide sequence of the SWP region among *M. rosenbergii* EHPs obtained in this study and other EHPs available in the GenBank database; Figure S2: Alignment of nucleotide sequence of the ITS-1 region among *M. rosenbergii* EHPs obtained in this study and other EHPs available in the GenBank database.
