# Supplementary material for: Detection and Genetic Characterization of Enterocytozoon hepatopenaei in Giant Freshwater Prawn (Macrobrachium rosenbergii) Imported into South Korea
Source: Animals (Basel). 2025 Nov 13;15(22):3286. doi: 10.3390/ani15223286 (PMC12649716; doi:10.3390/ani15223286)

Supplementary Table S1. Detailed information on the SSU rRNA, SWP 1, and ITS-1 sequences of *Enterocytozoon hepatopenaei* (EHP) obtained from this study.

Target gene	Sample ID	Accession no.	Size (bp)
SSU rRNA	23-026C6-1-VIE	PP212974	1031
	23-026C6-2-INDIA	PP212975	974
	23-026C7-1-VIE	PP212976	1031
	23-026C7-2-INDIA	PP212977	1024
	23-026C7-3-VIE	PP212978	1001
	23-026C9-2-VIE	PP212979	1028
SWP 1	23-026C6-1-VIE	PP908959	180
	23-026C6-2-INDIA	PP238911	180
	23-026C7-1-VIE	PP238912	180
	23-026C7-2-INDIA	PP238913	180
	23-026C7-3-VIE	PP238914	180
	23-026C9-2-VIE	PP238915	180
	23-026C11-1-VIE	PP238916	180
ITS-1	23-026C6-1-VIE	PP265522	436
	23-026C6-2-INDIA	PP265523	436
	23-026C7-1-VIE	PP265524	436
	23-026C7-2-INDIA	PP265525	406
	23-026C7-3-VIE	PP265526	322
	23-026C9-2-VIE	PP265527	409
	23-026C11-1-VIE	PP265528	409

Supplementary Table S2. Nucleotide sequence identities of the SSU rRNA region among *M. rosenbergii* EHPs obtained in this study and other EHPs available in the GenBank database.

	PP212974	PP212975	PP212976	PP212977	PP212978	PP212979	OP363710	MZ819965	KY643648	KX981865
PP212975	100									
PP212976	100	100								
PP212977	99.7	99.7	99.7							
PP212978	99.7	99.7	99.7	99.7						
PP212979	100	100	100	99.7	99.7					
OP363710	99.5	99.5	99.5	99.4	99.4	99.5				
MZ819965	99.7	99.7	99.7	99.6	99.6	99.7	99.7			
KY643648	99.9	99.9	99.9	99.7	99.7	99.9	99.6	99.9		
KX981865	99.9	99.9	99.9	99.7	99.7	99.9	99.6	99.9	100	
KP759285	99.9	99.9	99.9	99.7	99.7	99.9	99.6	99.9	100	100

Supplementary Table S3. Nucleotide sequence identities of the SWP 1 region among *M. rosenbergii* EHPs obtained in this study and other EHPs available in the GenBank database.

	PP908959	PP238911	PP238912	PP238913	PP238914	PP238915	PP238916	MZ541056	MH365434	MG015710	KY593133
PP238911	100										
PP238912	100	100									
PP238913	98	98	98								
PP238914	98	98	98	100							
PP238915	100	100	100	98	98						
PP238916	100	100	100	98	98	100					
MZ541056	97.4	97.4	97.4	99.3	99.3	97.4	97.4				
MH365434	97.4	97.4	97.4	99.3	99.3	97.4	97.4	100			
MG015710	97.4	97.4	97.4	99.3	99.3	97.4	97.4	100	100		
KY593133	97.4	97.4	97.4	99.3	99.3	97.4	97.4	100	100	100	
MW269619	90.7	90.7	90.7	90.1	90.1	90.7	90.7	90.7	90.7	90.7	90.7

Supplementary Table S4. Nucleotide sequence identities of the ITS-1 region among *M. rosenbergii* EHPs obtained in this study and other EHPs available in the GenBank database.

	PP265522	PP265523	PP265524	PP265525	PP265526	PP265527	PP265528	ON015652	OR162445	OR168076
PP265523	100									
PP265524	100	100								
PP265525	69.4	69.4	69.4							
PP265526	74.8	74.8	74.8	86.6						
PP265527	100	100	100	69.4	74.8					
PP265528	100	100	100	69.4	74.8	100				
ON015652	74.2	74.2	74.2	83.5	92	74.2	74.2			
OR162445	74.2	74.2	74.2	83.5	92	74.2	74.2	100		
OR168076	74.2	74.2	74.2	83.5	92	74.2	74.2	100	100	
MNPJ00000000	74.5	74.5	74.5	83.9	92.3	74.5	74.5	99.7	99.7	99.7

Supplementary Figure S1. Alignment of nucleotide sequence of the SWP region among *M. rosenbergii* EHPs obtained in this study and other EHPs available in the GenBank database.

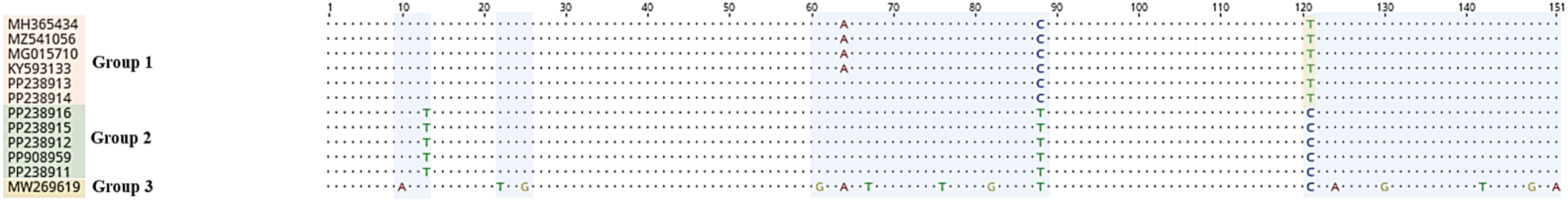

Supplementary Figure S2. Alignment of nucleotide sequence of the ITS-1 region among *M. rosenbergii* EHPs obtained in this study and other EHPs available in the GenBank database.

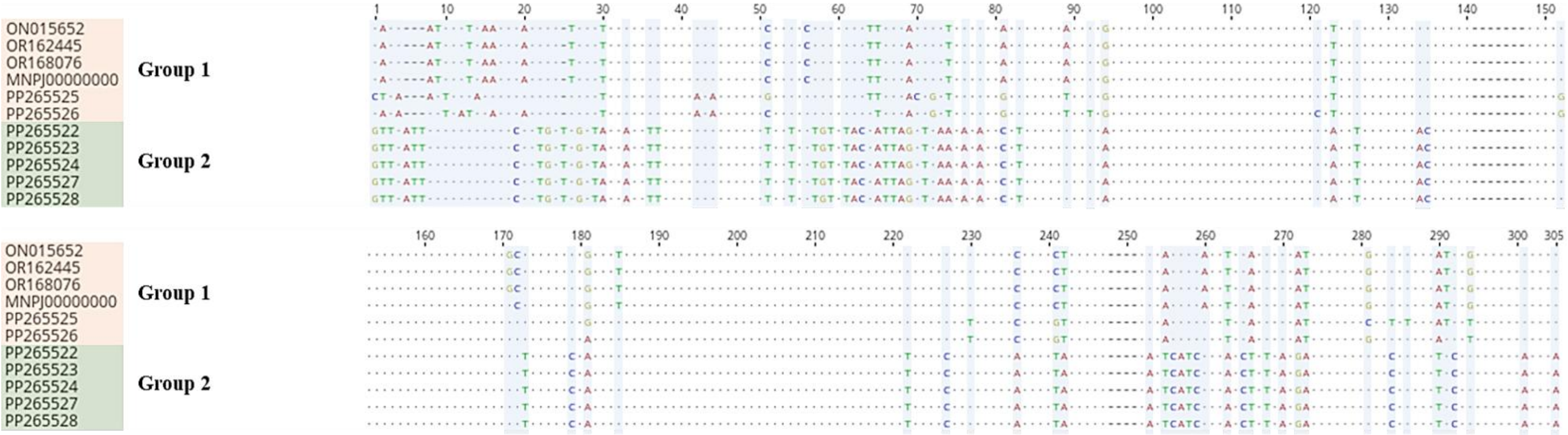

Supplement: Supplementary file 1 [file animals-15-03286-s001.zip › animals-3878727-supplementary.pdf]
